# Supplementary material for: Reward modulates the effect of visual cortical microstimulation on perceptual decisions
Source: eLife. 2015 Sep 24;4:e07832. doi: 10.7554/eLife.07832 (PMC4616243; doi:10.7554/eLife.07832)
Supplement: Supplementary file 1. — The nature of the reward contingencies in this experiment meant that the reward size for any particular trial was dictated by the monkey's accumulated performance on the previous trials. Therefore, the distribution stimulus disparities and the proportion of microstimulated trials were not a priori balanced between large and small reward conditions. Any systematic difference between the distributions of these factors in the two reward conditions might contribute to the reported effect of reward on microstimulation shift. We compared the mean absolute disparity and the percentage of microstimulated trials in small reward versus large reward trials. We found no significant difference in the distribution of stimulus disparities between the reward conditions in either monkey across all sites (Wilcoxon sign-rank test, Fle: n = 20, p = 0.263; Ica: n = 28, p = 0.187) nor when significant PREF sites are considered alone (Fle: n = 13, p = 0.127; Ica: n = 15, p = 0.169). We also found no significant difference in the proportion of microstimulated to non-stimulated trials in the two reward conditions in either monkey across all sites (Wilcoxon sign-rank test, Fle: n = 20, p = 0.737; Ica: n = 28, p = 0.075), nor when significant PREF microstimulation sites were considered alone (Fle: n = 13, p = 0.735; Ica: n = 15, p = 0.064). DOI: http://dx.doi.org/10.7554/eLife.07832.017 [file elife07832s001.docx]

**Additional file types: Supplementary file 1**

**Supplementary table 1. Large and small reward conditions do not differ in the proportion of microstimulated trials, nor in the distribution of stimulus disparities.**

|  | MEAN ABSOLUTE STIMULUS DISPARITY | | | % MICROSTIMULATED TRIALS | | |
| --- | --- | --- | --- | --- | --- | --- |
| **FLE** | **small reward trials** | **large reward trials** | **p-value** | **small reward trials** | **large reward trials** | **p-value** |
| sig. PREF sites | 0.022 | 0.024 | p = 0.127 | 0.5 | 0.5 | p = 0.735 |
| all sites | 0.018 | 0.019 | p = 0.263 | 0.5 | 0.5 | p = 0.737 |
| **ICA** |  |  |  |  |  |  |
| sig. PREF sites | 0.014 | 0.013 | p = 0.169 | 0.51 | 0.49 | p = 0.064 |
| all sites | 0.010 | 0.010 | p = 0.187 | 0.51 | 0.49 | p = 0.075 |
